# Pre-service Teachers' Use of General Social Networking Sites Linked to Current Scenarios: Nature and Characteristics

**DOI:** 10.1007/s10758-022-09609-7

**Published:** 2022-05-23

**Authors:** Diego Calderón-Garrido, Raquel Gil-Fernández

**Affiliations:** 1 Serra Húnter Fellow, Department of Applied Didactics, Faculty of Education, Universitat de Barcelona de la Universidad de Barcelona, Passeig de la Vall d’Hebron, 171, 08035 Barcelona, Barcelona Spain; 2 Department of Didactics of Social Sciences, Faculty of Education, Universidad Internacional de la Rioja, Av. de la Paz, 137, 26006 Logroño, La Rioja Spain

**Keywords:** Social media, Higher education, Teachers degrees, Pre-service teachers

## Abstract

Social networking sites form part of everyday life in classrooms at all educational levels. Within these, general social networking sites (GSNSs) offer pre-service teachers flexibility, versatility and the possibility of forming educational communities by connecting formal, non-formal and informal settings. This research analyses the nature, intensity, and type of pre-service teachers’ use of such for educational purposes in their initial training in order to detect the most important aspects for improvement. Possible factors shaping behaviour were gender, whether individuals belonged to universities operating online or in person, differences in the types of studies they were undertaking, and the time at which the questionnaire was administered, before or after the COVID-19 health crisis. To this end, we studied how much and with what aims these students use the most widely used GSNSs for educational purposes. To do so, we administered a questionnaire to a total of 812 students from 6 Spanish universities. The results show a preference for WhatsApp, YouTube, and Instagram. In addition, it was found that undergraduate students used them more intensively than postgraduate students. In the case of online universities, there was a greater need to cover affective and emotional aspects than in in-person universities. As in almost all areas, the situation caused by COVID-19 changed the way social networks were used. The findings also show that pre-service teachers consumed more information on social media than what they produced, which leads to a failure to fully exploit social capital and potential job or academic opportunities that could be generated through their own creations.

## Introduction

Social networking sites form part of everyday life in classrooms at all levels of education. This has resulted in a clear paradigm shift caused by the appearance of these networks in educational settings (Amer & Amer, 2018). For university students, social networking sites provide a link between the classroom and the world outside it. Consequently, there is a need to explore how they shape the ways they use social networking sites. In this case, we consider pre-service teachers and how they connect in educational environments. These networks offer future teachers the opportunity to self-manage their learning (Durak, 2019) and enhance and strengthen their connections with the educational community and interaction in the learning process (Torphy et al., 2020a, b, c). Nowadays, everyday knowledge is generated through social media such as Facebook, YouTube, WhatsApp, WeChat and Instagram, which connect teachers with other people and promote group work and interactivity, thus forming a social identity (Khoza, 2021).

This research analyses the nature, intensity, and type of educational use,made of these networks by students studying for bachelor’s degrees in early childhood education and in primary education, the double degree in early childhood and primary education, and the master’s degree in secondary education. To this end, we studied how much and with what aims these students use the most widely used general social networking sites (GSNSs) for educational purposes.

In an educational context, social networks enable certain formal constraints on learning to be removed and have characteristics that include visibility, transparency and the possibility of creating open communities (Hatzipanagos & Warburton, 2009).

To achieve this objective, we have considered various aspects and factors that shape behaviour, such as whether the individuals we consulted attended universities that operate online or are brick-and-mortar institutions, differences between types of programmes, and the differences in the use of GSNSs before and after the start of the COVID-19 crisis.

Therefore, our research questions are as follows:

General RQ: What is the nature, intensity and type of educational use made of GSNSs by pre-service teachers?

RQ1: What are the most widely used GSNSs in the population analysed?

RQ2: Are there any gender differences?

RQ3: What are the differences between undergraduate and postgraduate students regarding use?

RQ4: Are there any noticeable differences between students at in-person universities and universities operating exclusively online?

RQ5: What differences have been established between pre- and post-pandemic uses?

RQ6: Which specific educational uses are most common, and which are still in the process of implementation?

The uses revealed by the sample and their subsequent grouping into categories guide the review of academic literature that forms the basis of this paper, with the most important concepts set out below.

## Theoretical Framework

There is a vast theoretical foundation for analysing the use of educational technology in general and of social networks in particular. Davis (1986) based his work on the Theory of Reasoned Action (TRA) by Fishbein and Ajzen (1975) and formulated the technology acceptance model (TAM; Althunibat, 2015; Camilleri & Camilleri, 2021; Park et al., 2007). However, the flaws in this TAM were identified by its own creator, who discovered that it lacked the variables to answer questions such as perceived usefulness or ease of use. Consequently, in order to find a more contextual explanation, he supplemented his model with the approach of the uses and gratification theory (UGT) (Katz et al., 1974), which established a valid theoretical model to understand the reason behind the choice (Saini & Abraham, 2019). This model consists of five items: perceived ease of use, perceived usefulness, attitudes towards technology, the intention to use it and real behaviours (Al-Rahmi, 2021; Camilleri & Camilleri, 2021; Davis, 1989).

Another theory based on the TRA is the theory of planned behaviour (TPB; Park et al., 2012), which has been widely used. Venkatesh et al. (2012) used it to propose the unified theory of acceptance and use of technology (UTAUT).

Al-Rahmi et al. (2021) used the information system success model (ISSM) in conjunction with the TAM to be able to explore students’ behaviour when using social networks for educational purposes and to determine their perception of their own academic performance, as well as their satisfaction. Al-Maatouk et al. (2020), also used the task-technology fit (TTF) model to analyse the use of networks, as this model takes into account the technology, task and social characteristics.

### Establishing Learning Communities

Social networking sites are a factor and vector for the optimal inclusion of the people who will educate and who are learning how to educate future citizens in the framework of a society undergoing constant change, as suggested by Barak (2017). In recent years, research has always been based on the premise of not restricting teachers’ autonomy or shaping their creativity, regardless of the perspective (McGarr & McDonagh, 2020).

Scientific literature has proven the value of community-based learning, whether in traditional, blended, or online environments (Jan & Vlachopoulos, 2019). Social networks have been used to create influence networks and find support and professional mentoring (Minor et al, 2019). In this way, social networking sites form real learning communities and meaningful education networks, whether between peers (teachers or students) or between teachers and students, as pointed out by several authors (Carpenter et al., 2020; Chang-Kredl & Colannino, 2017; Dennen et al., 2020; Greenhalgh et al., 2020; Hasiloglu et al., 2020; Wickramanayake & Jika, 2018). This, in turn, has a beneficial impact on the teaching–learning process (Torphy et al., 2020a, b, c).

GSNSs are characterised by their lack of delimitation and specificity, which aids their flexibility of use and possibilities of adapting to the user's aims. Hatzipanagos and John (2017), after comparing the use of an institutional social network with the general ones—called “commercial” or “public” by the authors, found that users of the former valued the more professional and academic aspect that it offered, as well as aspects related to privacy and security. However, the results also revealed that most students did not use it, or did not even know about it, thus favouring the use of GSNSs because of their greater versatility, popularity, ease of access, functionality, and the possibility of interacting with groups outside of the university itself.

Therefore, a number of authors regard GSNSs as an excellent catalyst for the sharing of ideas and connecting formal and non-formal settings, sometimes on a par with excessively rigid hierarchical structures to achieve more ecological and sustainable models (Kamalodeen & Jameson-Charles, 2016; Torphy et al., 2020a, c; Carpenter & Harvey, 2019).

However, there are gaps in the study of the emotional dimension relating to personal accompaniment in order to prevent professional isolation (Staudt Willet, 2019). According to Ekoç (2020), this is somewhat of a paradox as social networking sites are actually intended to prevent this very isolation, and in studies maintained over time, such as that by Chen et al. (2019), the positive outcome in terms of emotional development and sense of belonging can be seen. Carpenter et al. (2019) studied the relationship between personal and professional identity. They concluded that there is an imbalance, as the personal information the group shares on GSNSs is limited and impersonal. In this sense, Tyrer (2019) also notes the importance of vertical moral support, flowing from more experienced individuals to newcomers.

As regards motivational aspects, academic literature has taken an interest in topics such as self-disclosure on social networking sites (Imlawi et al., 2015), empowerment through motivation, and students’ degree of satisfaction (Trust, 2017). In the case of teachers, Hashim and Carpenter (2019) determined the range of motivational factors that lead teachers to establish and design their online practices. Similarly, the “motivational influence” construct is the key factor for predicting the choice and use of specific networks for Saini and Abraham (2019).

Another dimension where authors agree that it is necessary to make progress is the issue of critical thinking in teachers. For example, the study by Santisteban et al. (2020) identified the difficulties in establishing critical discourses in future teachers when interpreting the information they posted on social networks. A predictive study on this matter is, therefore, required.

With regard to pre-service teachers in their initial training, Brouwer et al. (2020) evaluated the relationships between them maintained through social networks. Studying these relationships made it possible to establish a sequence in which people first seek to make friends, then establish communication between peers, and finally receive professional guidance with very positive results.

Therefore, it is important to note that one of the most important uses when managing learning through GSNSs is the acquisition of the social capital created in these settings, and this is one of the most important outcomes (Goodyear et al., 2019). Similarly, one dimension to consider about the acquisition of social capital is the perception of self-efficacy, social support, and collegial practices (Lantz-Andersson et al., 2018; Rensfeldt et al., 2018).

### The Different GSNSs as a Vehicle for Teaching Resources in the Teaching–Learning Process

The idea of obtaining content from GSNSs in order to transfer resources from certain platforms, obtaining or adapting teaching resources, and managing content on them, has shaped many practices and experiences in the work of pre-service teachers (Goodyear et al., 2014). Consequently, their asynchronous nature and lack of spatial limits are the main attractions of these networks. However, although universities currently offer all sorts of media and collections of resources on their own institutional platforms, teaching staff and students still continue to use GSNSs to provide experiences and locate resources (Becerra & Martín, 2015).

For example, some networks are used extensively by teachers to find resources as they are easy to search and versatile and due to their possibilities to create resources, as is the case with YouTube (Barry et al., 2016; Chintalapati & Daruri, 2017). The networks used most to structure emerging practices for cooperative learning are Facebook and Twitter (Tur et al., 2017). These networks also present proposals based on continuous assessment (McCarthy, 2017).

In the case of Twitter, Pérez-Garcías et al. (2020) concluded that it could be a good resource for pre-service teachers as the limited number of characters obliges users to reflect and choose their words carefully. With regard to Facebook, Thompson et al. (2015) proposed multiple practices for improving and reinforcing knowledge. Other experiences were based on interactive contexts, placing value on dialogue (Simpson, 2016). Valid metaphors have also been considered (Krutka et al., 2014) and experiments have been performed with a view to future teachers acquiring particular skills in order to foster self-directed learning (Carpenter & Krutka, 2015; Staudt Willet, 2019).

However, in the case of the usability of social networking sites, Durak (2019) was concerned about self-management—a very important dimension—and she warns of the limited use of social networking sites in teacher training. Accordingly, although the use of social networking sites is a key competence for the twenty first century, it is believed that teachers receive insufficient training in this area (Sinnema et al., 2020). Prestridge et al. (2019) note this inconsistency and suggest good practices for remedying it and developing teachers' experience. In their paper, they emphasised the possible transformation of the concept of “sharing”, given the implications of GSNSs as a source of learning.

### Limitations in the Didactic Use of GSNSs

There are numerous studies linking teachers’ digital competence to the use of social media, many of which conclude that a lack of teacher training in this regard (Barak, 2017; Durak, 2019) leads to a failure to fully exploit valuable resources (Sinnema et al., 2020). Some papers analyse the educational use of social networks by means of the UGT (Gil-Hernández & Calderón-Garrido, 2021), often combined with the Social Network Theory (Yakin & Tinmaz, 2015), the Decomposed TPB (Dermentzi et al. 2016), and the MAIN model (Rathnayake & Winter, 2018). In some cases, negative effects are reported due to the excess time spent on social media compared to its benefits (Mingle & Adams, 2015); in other cases, the need is revealed for universities to make inclusive decisions to increase the educational use of social networks, as it is considered to be very limited (Dertmentzi, 2016).

Optimal use of social networks requires critical thinking skills. In this regard, Santisteban et al. (2020), measured the degree of acquisition of such skills in pre-service teachers and the results revealed difficulties being critical due to deficiencies in argumentative skills. Greene (2017), despite trusting in the role of digital narratives, warned of the need for an in-depth critical analysis. Despite understanding the appeal of online communities, Matzat (2013) analysed mistrust between peers and the feeling of people taking advantage that occurs in online settings and recommended that they be complemented by in-person meetings. Dhir et al. (2017) investigated teachers' mistrust of social networks and their use in the classroom, which often leads to minimal use. Ghareb et al. (2018) warn about GSNSs, highlighting that they sometimes host inappropriate content for education, and that they lack certain features, as do Kim & Malek (2018), who also considered the disadvantages.

Regarding adverse effects on emotional aspects, Raza et al. (2020) note benefits and improvements in the lives of students using GSNSs, but also warn of the danger of “social overload”. Marín et al. (2020) addressed the issue of privacy and concluded that although pre-service teachers were aware of the teaching potential of social networks, they sometimes lacked knowledge about data privacy policies and regulations, due to a lack of literacy in this area.

## New Scenarios, New Challenges

The outbreak of COVID-19 and the rapid changes made in the different educational environments have given rise to “emergency remote teaching” (Hodges et al., 2020). This has affected all areas of relationships and, naturally, virtually all the variables analysed have been affected by the pandemic. It cannot be denied that all universities turned to digital technologies to be able to continue teaching their students. In fact, as a result of many of the changes that took place, the possibility of including real-time interactive communication emerged. In this way, both teachers and students would be able to continue the teaching process (Camilleri, 2021). The facilitating conditions of both teachers and institutions were key to achieving this (Camilleri & Camilleri, 2022). According to Teräs (2020), education during COVID-19 revealed five principles for guaranteeing the success of online university education and the use of educational technologies. These same principles shaped the post-pandemic period: learning content had to correspond to behaviour and teaching preparation, pacing was vital to avoid a lack of concentration, assistance had to be provided and clear instructions given, student participation had to be encouraged and there had to be provisions to deal with any technical problems.

In this respect, it is important to note that it has resulted in a series of changes in both the intensity of use of GSNSs and how students relate with one another, especially at times when there were full lockdowns in different countries (Gil-Fernández et al., 2021). The online classes taught via GSNSs, apart from their educational aspect, provided social support, revealing their more emotional side and aiding not only learning but also psychological resilience (Ashgar et al., 2021).

García-Peñalvo et al. (2020) pointed out that the emergency actions taken in the first instance cannot be claimed to be analogous to proposals specifically designed for online delivery, but also noted that there has been an acceptable transition to online classes in in-person universities, which has yielded good results.

The introduction, maintained over time, of a greater use of social networks in the educational environment requires preventing the risk of reproducing declarative and passive patterns on social networks (Abreu, 2020). Therefore, the main challenge involves not only introducing technologies, but making profound changes (Barrón, 2020) that affect the use of social networks. Authors such as Cavus et al. (2021) and Greenhow and Chapman, (2020) reveal the need for effective strategies for sustainable educational use of social networks involving institutions, teachers and learners.

## Empirical Context and Methodology

### Empirical Context

To achieve the proposed objectives of this paper, we created and then administered an online questionnaire considering the specific characteristics of students studying for bachelor’s degrees in early childhood and primary education, the double degree in early childhood and primary education, and the master’s degree in secondary education. We chose the study population at random from different subjects in different years from six Spanish universities, four of them brick-and-mortar institutions (Universidad de Barcelona, Universidad de Sevilla, Universidad de Cantabria, and Universidad Jaume I) and two online (Universidad Internacional de la Rioja and Universidad Nacional de Educación a Distancia). These universities were chosen because they were the ones to which the researchers had direct access. Students were contacted through institutional email addresses, reaching a potential sample of 3000 students. Out of this potential sample, 876 people responded. After cleansing the data, the sample analysed contained a total of 812 students who had fully answered the questionnaire. We administered the questionnaire in two different periods: the first before the COVID-19 crisis (potential sample of 1996 students) and the second during said crisis (potential sample of 1004 students). Out of this potential sample, 876 people responded. It is a non-probabilistic convenience sample. Table 1 shows all of the characteristics of the sample.

**Table 1:** Sample by age, gender, studies, and type of university

		Age	Gender		Degree				Type	
			Male	Female	Childhood	Primary	Double	Master’s	In person	Online
Age	29.92 SD = 8.47									
Gender										
Male	166 20.4%	31.27 SD = 8.87								
Female	646 79.6%	29.57 SD = 8.34								
Degree										
Childhood	259 31.9%	29.54 SD = 8.56	9 3.5%	250 38.7%						
Primary	332 40.9%	29.36 SD = 8.46	73 22%	259 40.1%						
Double degree	34 4.2%	23.91 SD = 7.12	4 11.8%	30 4.6%						
Master’s	187 23%	32.53 SD = 7.81	80 42.8%	107 16.6%						
Type										
In person	224 27.6%	22.08 SD = 5.79	38 22.9%	186 28.8%	72 32.1%	112 50%	15 6.7%	25 11.2%		
Online	588 72.4%	32.91 SD = 7.35	128 77.1%	460 71.2%	187 31.8%	220 37.4%	19 3.2%	162 27.6%		
Lockdown										
Pre	448 55.2%	32.79 SD = 7.78	105 23.4%	343 76.6%	123 27.5%	160 35.7%	11 2.5%	154 34.4%	34 7.6%	414 92.4%
COVID	364 44.8%	26.39 SD = 7.95	61 16.8%	303 83.2%	136 37.4%	172 47.3%	23 6.3%	33 9.1%	190 52.2%	174 47.8%

### Materials

In terms of the analytical procedure, when designing the questionnaire we consulted a panel of four experts in educational technology and used the Delphi method (Somerville, 2008). We analysed their feedback using Fleiss’ kappa (.89) and Kendall’s W (.87), which gave values with relevance and pertinence among the opinions. In addition to the structure of the questionnaire that would later be used, this process resulted in the GSNSs to be explored (Facebook, Instagram, Pinterest, Skype, SoundCloud, TikTok, Tumblr Twitter, WhatsApp and YouTube).

The final questionnaire, “Use of social networking sites in initial teacher training”, (available at https://reunir.unir.net/123456789/6695) comprised 6 demographic questions and 21 questions relating to the use of social networking sites. Out of these, 10 referred to the regular use of certain GSNSs. These were dichotomous. A further 10 questions asked about the academic use of these GSNSs. These questions used a four-level Likert-type scale. The last one was an open-ended question.

We used Bartlett’s test of sphericity to check dependence between the variables of the questionnaire (2586.41; p < 0.001) and the Kaiser–Meyer–Olkin test to check the adequacy of the sample (KMO = 0.83). The questions referring to the use of GSNSs displayed excellent consistency and internal reliability (Cronbach’s alpha = .88).

### Procedure

We administered the questionnaire online using the Formsite platform, allowing a period of three weeks for responses in both cases. The students who participated gave their free, prior and informed consent which was explained in the questionnaire itself. They were informed that they could withdraw from the study at any time.

### Data Analysis

We used the IBM Statistical Package for the Social Sciences (SPSS) program, version 21.0, to calculate and statistically analyse the results. In all cases, a minimum confidence interval of 95% was established. The significance threshold for all tests was set at p < .05 (two-tailed). We used the statistical tests Mann–Whitney for two variables and Kruskal–Wallis for more than two variables to find statistically significant differences, having first applied the Kolmogorov–Smirnov or Shapiro–Wilk sample normality tests as required, as well as Levene’s test for equality of variances. We used the ATLAS.ti program, version 1.5.2, for the qualitative analysis of the answers.

## Findings

The results we obtained are shown below. As Table 2 shows, the most commonly used social networking sites are WhatsApp (96.3%), YouTube (94.2%), and Instagram (72.4%). However, regarding their educational use, and having sorted those who use each social networking site, we found that the most used are YouTube (60.9% of respondents use it often or always), Pinterest (55.3%), and WhatsApp (48.8% use it often or always).

**Table 2:** Results obtained

	Commonly used		Educational use			
	No	Yes	Never	Sometimes	Often	Always
Facebook	270 33.3%	542 66.7%	170 31.4%	214 39.5%	122 22.5%	36 6.6%
Instagram	224 27.6%	588 72.4%	242 41.2%	194 33%	101 17.2%	51 8.7%
Pinterest	369 45.4%	443 54.6%	64 14.4%	134 30.2%	155 35%	90 20.3%
Skype	374 46.1%	438 53.9%	165 37.7%	165 37.7%	77 17.6%	31 7.1%
SoundCloud	732 90.1%	80 9.9%	37 46.3%	27 33.8%	13 16.3%	3 3.8%
TikTok	583 71.8%	229 28.2%	177 77.3%	40 17.5%	12 5.2%	0
Tumblr	764 94.1%	48 5.9%	27 56.3%	17 35.4%	2 4.2%	2 4.2%
Twitter	421 50.8%	391 48.2%	133 34%	156 39.9%	71 18.2%	31 7.9%
WhatsApp	30 30.7%	782 96.3%	126 16.1%	267 34.1%	253 32.4%	136 17.4%
YouTube	47 5.8%	765 94.2%	38 5%	261 34.1%	377 49.3%	89 11.6%

Regarding the educational use of the different social networking sites, as Table 3 shows, we found statistically significant differences according to gender in the case of Facebook (*Z* =  − 4.78, *p* < .001), Instagram (*Z* =  − 3.72, *p* < .001), Pinterest (*Z* =  − 3.69, *p* < .001), WhatsApp (*Z* =  − 4.78, *p* = .001), and YouTube (*Z* =  − 2.43, *p* = .015). In all cases, women were more likely to use them than men.

**Table 3:** Statistical differences and means by gender

		Facebook	Instagram	Pinterest	Skype	SoundCloud	TikTok	Tumblr	Twitter	WhatsApp	YouTube
Statistical differences	*Z*	− 4.78**	− 3.72**	− 3.69**	− 1.13	− 1.37	− 1.24	− .17	− .61	− 3.22*	− 2.43*
Male	*x* *SD*	.71 .90	.66 .94	1.10 .95	.84 .85	.53 .69	.25 .72	.53 .62	1.05 .94	1.29 .98	1.53 .75
Female	*x* *SD*	1.13 .87	.99 .96	1.68 .95	.97 .93	.85 .89	.30 .57	.58 .85	.98 .91	1.57 .95	1.71 .74

**p < .00; *p < .05

With regard to differences according to the degree programme, we observed statistically significant differences in the case of Facebook [*X*^2^_(3)_ = 41.51, *p* < .001], Instagram [*X*^2^_(3)_ = 17.18, *p* = .001], Pinterest [*X*^2^_(3)_ = 21.38, *p* < .001], and Skype [*X*^2^_(3)_ = 11.45, *p* = .010]. When dividing the sample between the bachelor’s degrees and the master’s degree, we observed statistically significant differences in the case of Facebook (*Z* =  − 4.69, *p* < .001), Instagram (*Z* =  − 3.66, *p* < .001), Pinterest (*Z* =  − 4.23, *p* < .001), Skype (*Z* =  − 3.69, *p* = .007), and SoundCloud (*Z* =  − 1.99, *p* = .047). In all cases, undergraduate students used social networking sites more than master’s students. Table 4 shows these data.

**Table 4:** Statistical differences and means by bachelor’s and master’s degrees

		Facebook	Instagram	Pinterest	Skype	SoundCloud	TikTok	Tumblr	Twitter	WhatsApp	YouTube
Statistical differences	*X*^2^_(3)_	41.51**	17.18*	21.38**	11.45*	6.63	5.97	5.25	6.63	7.67	1.53
Childhood	*x* *SD*	1.33 .83	1.09 .97	1.80 .90	.83 .79	.95 .78	.18 .51	.29 .76	.81 .83	1.64 .94	1.66 .71
Primary	*x* *SD*	.96 .84	.90 .91	1.67 .92	1.12 1.01	.76 .90	.30 .54	.62 .80	.81 .83	1.64 .94	1.66 .71
Double degree	*x* *SD*	1.58 1.12	1.03 .99	1.39 1.19	1.20 1.08	1.40 1.14	.44 .53	1.46 .92	1.00 1.08	1.61 .90	1.70 .75
Master’s	*x* *SD*	.77 .90	.69 1.02	1.18 1.02	.75 .77	.45 .69	.75 .96	.47 .51	1.08 .93	1.41 1.03	1.64 78
Statistical differences	*Z*	− 4.69**	− 3.66**	− 4.24**	− 2.69*	− 1.99*	− 1.47	− .036	− 1.12	− 1.69	− 2.64
Degree	*x* *SD*	1.15 .87	.99 .94	1.71 .93	1.03 .96	.88 .88	.25 .52	.62 .90	.97 .91	1.54 .94	1.69 .73
Master’s	*X* *SD*	.77 .89	.69 1.02	1.18 1.03	.75 .77	.45 .69	.75 .96	.47 .51	1.08 .93	1.41 1.03	1.64 .78

**p < .00; *p < .05

With regard to the type of university, as shown in Table 5, we observed statistically significant differences regarding the use of Facebook (*Z* =  − 3.63, *p* < .001) and Twitter (*Z* =  − 2.00, *p* < .045). These were more commonly used in online universities. We also observed statistically significant differences with Skype (*Z* =  − 7.39, *p* < .001) and SoundCloud (*Z* =  − 4.01, *p* < .001), which were used more at brick-and-mortar universities.

**Table 5:** Statistical differences and means by type of university

		Facebook	Instagram	Pinterest	Skype	SoundCloud	TikTok	Tumblr	Twitter	WhatsApp	YouTube
Statistical differences	*Z*	− 3.63**	− .409	− 1.21	− 7.39**	− 4.01**	− .29	− .66	− 2.00*	− 4.15**	− 2.04*
In-person	*x* *SD*	.74 .82	.91 .85	1.52 .93	1.52 .94	.15 .37	.28 .54	.50 .86	.88 .92	1.73 .85	1.61 .79
Online	*x* *SD*	1.10 .90	.94 1.02	1.64 .98	.75 .82	.98 .87	.27 .65	.59 .74	1.06 .91	1.43 .99	.76 .65

**p < .00; *p < .05

With regard to use of social networking sites before and after the lockdown caused by COVID-19, as shown by Table 6, there were statistically significant differences in the cases of Instagram (*Z* =  − 2.05, *p* = .040) and Skype (*Z* =  − 6.77, *p* < .001), with both being used more during the COVID-19 period. We also observed differences in the cases of SoundCloud (*Z* =  − 2.40, *p* = .016), Twitter (*Z* =  − 2.02, *p* = .043), and YouTube (*Z* =  − 3.605, *p* < .001), which were used more prior to COVID-19.

**Table 6:** Statistical differences and means pre-COVID and during COVID

		Facebook	Instagram	Pinterest	Skype	SoundCloud	TikTok	Tumblr	Twitter	WhatsApp	YouTube
Statistical differences	*Z*	− 1.40	− 2.05*	− .77	− 6.77**	− 2.40**	− .43	− .66	− 2.02*	− 1.86**	− 3.60**
Pre-COVID	*x* *SD*	1.08 .92	.89 1.04	1.58 .95	.72 .78	.91 .86	.23 .62	.63 .83	1.08 .93	1.46 1.02	1.76 .76
COVID	*x* *SD*	.96 .83	.98 .87	1.66 .99	1.38 .99	.46 .77	.28 .55	.44 .63	.89 .88	1.57 .89	1.58 .71

**p < .00; *p < .05

With regard to the open question about the use of social networking sites in education, by analysing the answers we were able to identify 12 patterns. As a result, we found that pre-service teachers who use these social networking sites, whether in individual work or group work, do so primarily to search for information and teaching resources. This also presents possible solutions relating to continuous training. For example, this is the case of Respondent 412 who, referring to the teaching practice period, reported using GSNSs “to find activities and resources to help me give the children a wider range of knowledge using methodologies I did not cover on my course. I also look for videos and information for students who find studying hard.”

Along the same lines, we also observed several references to self-learning. This is the case of Respondent 14 who said, “I look for songs that I can use in my teaching practice, and I learn them so that I can teach them to the students.”

In the case of group work and creating environments, respondents also used social networking sites for sharing information and establishing dialogue between the different members of each group. This is a constant in the case of online students, since, as Respondent 367 observed, “as we live far apart, we do not have contact with each other and so we use WhatsApp to help as much as we can and share material.”

We also observed many answers that mentioned following experts and important figures in the field, as well as the different public administrations in order to stay informed of current developments in education. This is the case of Respondent 84, who said, “above all, I use the Instagram app to follow accounts relating to education so that I am aware of news, courses, conferences, etc.”

Finally, there were several references to the motivational element of digital competence, which leads to the inclusion of ICT, and in the case of social networking sites, of different educational processes. For example, Respondent 702 was of the opinion that “for me it is much better to teach children, do games… using new technologies rather than flashcards. Therefore, I use networks and ICT quite a lot.”

**Fig. 1:**
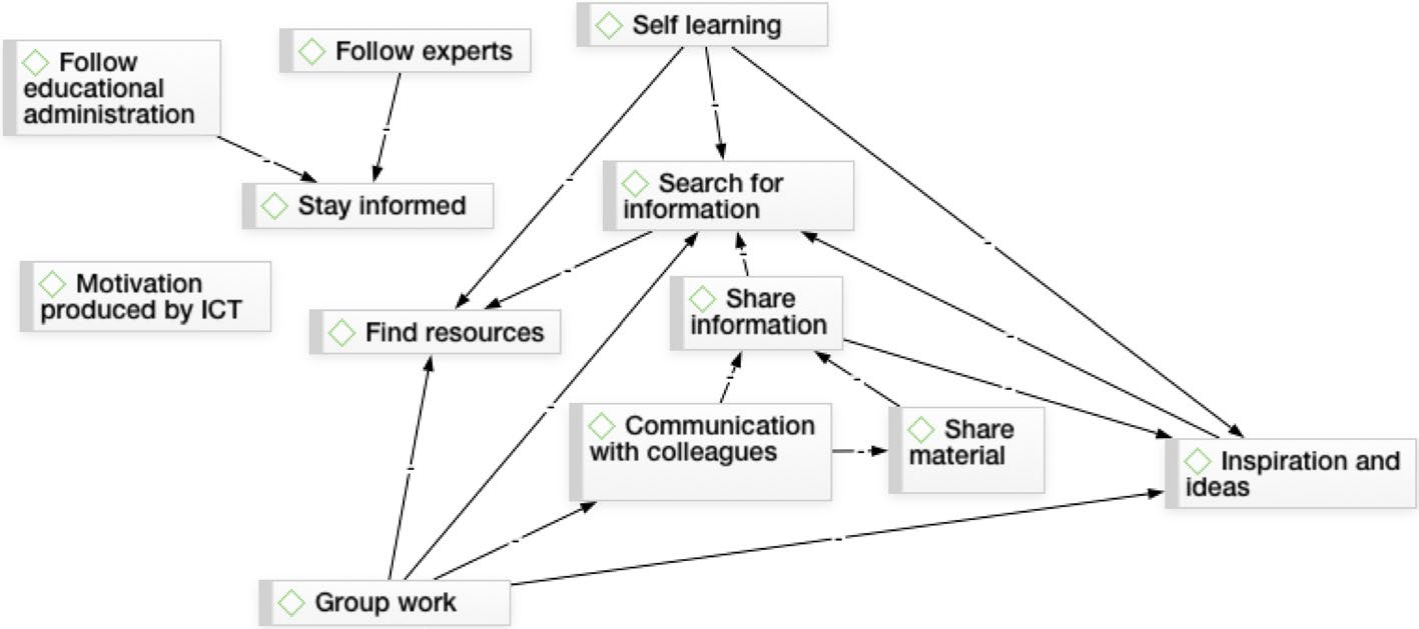
Co-occurrences in the use of social networking sites in educational settings

Figure 1 shows a summary of the different codes described and how they relate to one another.

## Discussion

Table 2 shows the most used networks. According to the data, the networks most commonly used and for general purposes were WhatsApp, YouTube, and Instagram, followed to a lesser extent by Pinterest and Skype. YouTube, Pinterest, and WhatsApp were used the most for teaching purposes.

WhatsApp was the most used network in the everyday life of the students in the sample. It was also one of the most widely used for educational purposes. Students reported using this GSNS to communicate in order to obtain quick feedback so that they could effectively resolve peer-to-peer queries. This idea is in line with multiple research papers on this network (Brouwer et al., 2020; Fuchs, 2019; Suárez, 2018 among others). Undoubtedly, the key aspects are its effectiveness, immediacy and cost (Fondevila, et al., 2019). Regarding the use of WhatsApp, the results reveal motivations already highlighted by other researchers (Tyrer, 2019): it is a network that, in addition to its immediacy, provides students, especially the less experienced ones, with a sense of companionship and moral and academic support among peers which is what leads to this GSNS forming part of the everyday life of the population studied. Studies such as the one undertaken by Annamalai (2018) reveal that pre-service teachers perceive this network as an ideal instrument for achieving small-scale learning with immediacy and seamlessness, which sometimes clashes with the opinion of their trainers, who are not very inclined to interact in this environment.

Szeto et al. (2016) observed that YouTube is very often used by students, in particular pre-service teachers, as they use it above all as a collection of teaching resources. This view is supported in the paper by Nackak et al. (2020), where the pre-service teachers in the sample pointed out the suitability of this network for finding resources, even mentioning dangers related to addiction to it, but at no point did they address the issue of representing the role of producers. It is a fact that YouTube is an excellent tool for innovation and creating one’s own content (Chintalapati & Daruri, 2017). However, teachers make virtually no use of its social aspect (Marchetti & Valente, 2018), thus missing out on some of the professional, social capital-related or emotional benefits described in the introduction explaining the theoretical background of this paper. This is also the case for the students in the sample related to this paper.

Pinterest was also identified as a resource pre-service teachers frequently use (Sawyer et al, 2019). This network is described as an ideal platform for finding educational resources (Schroeder et al., 2019) and also for displaying one’s own resources (Amer & Amer, 2018). Again, the need for critical literacy in order to make the best use of the resource is noted, as Schroeder et al. (2019) compared the use of this network between pre-service and in-service teachers and concluded that in-service teachers experienced more complex interaction by better detecting quality experiences.

Therefore, the most intense educational use that has been found is focused on resolving doubts and making social contact between peers (WhatsApp), watching videos/tutorials (as mere spectators and not producers) (YouTube), or following professionals or academic events related to education (Twitter). Thus, once again, it is clear that little use is made of the various features of the networks. This has been reported on various occasions (Köseoğlu & Köksal, 2018; Mnkandla & Minnaar, 2017), but does not appear to be improving.

With regard to differences by gender, it was found that women made greater use of Facebook, Instagram, Pinterest, WhatsApp, and YouTube than men. There are population-wide studies on gender differences in the use of social networks in education that coincide with the data from this study: women make greater use of GSNSs and use them for more functional purposes, including certain educational uses (Valencia-Ortiz et al., 2020).

With regard to differences between level of study, our results show that undergraduate students use social networking sites for educational purposes considerably more than master’s students in all cases. This is in line with previous research (Goodyear et al., 2014; Carpenter et al., 2016). We should not forget that social networking sites prevent isolation among peers and encourage motivation (Saini & Abraham, 2019). Therefore, this difference could be due to the very nature of the studies. Students on undergraduate courses have more time to establish human connections and build learning environments, and they can also maintain this contact over time as they spend longer at university. Master’s students, however, spend a shorter time at university. We also observed how master’s students opted for networks that made it possible to look for resources at the expense of ones with a more social dimension.

With regard to the type of university, we observed several differences. In online universities, in terms of the social element, there is evidence of a need to cover affective needs in the absence of physical contact (Colás et al., 2015). With regard to Facebook, this is in line with the findings of Phua et al. (2017), who also reported that this was more common among students aged over 30, as we observed in the sample we analysed (see Table 1). With regard to Twitter, we noted a certain parity of use, although it was also more common in online settings. However, despite the very positive conclusions of Canaleta et al. (2014) on its benefits to reflexive competences, it continues to be less often used.

With regard to brick-and-mortar universities, we observed more use of Skype. This could be because online universities usually have their own communication and video conferencing platforms that students use, while at brick-and-mortar universities they look for alternative routes. SoundCloud turned out to be used most in brick-and-mortar universities. This is in line with the data obtained by Nwosu et al. (2017), which showed more use of these types of voice notes in brick-and-mortar universities than in online ones, which favour the use of platforms involving the use of audiovisual files. There are studies that highlight how pre-service teachers show greater development of emotional and socio-educational skills than cognitive skills in their performance on social networks (Sánchez et al., 2017), which explains their increased use in purely online environments so as to prevent the isolation that can sometimes occur in this area. In short, it can be said that students studying at online universities make similar instrumental educational use of such networks but develop peer relationships more intensively than at in-person universities, taking advantage of the more “social” aspect of the networks.

With regard to the changes caused by COVID-19, as Camilleri and Camilleri (2021) explain, previous research was already reporting a generalised use of digital technologies in non-real time communications. However, it was during lockdown that students’ perceived usefulness, perceived interactivity, attitudes towards use, facilitating conditions and behavioural intentions and, ultimately, the usefulness of digital technologies changed significantly.

Rogers et al. (2020), indicated that there have been profound changes at a global level with evidence of variations in the temporality and likelihood of certain habits relating to GSNSs (Hodges et al., 2020). This has resulted in intensive use of GSNSs in both educational and personal environments, with the perception of the digital world changing drastically (Chamarro, 2020). This circumstance is in line with some isolated data obtained in this research, such as those of Skype. This network has established itself as a temporary support as it is free and multi-platform and is easy to configure, which gives it an advantage for simultaneous work (2020). This was also the case for Instagram, the use of which increased during the pandemic, despite it already being one of the students’ favourite networks owing to its versatility and range of image management options (Peña et al., 2018). WhatsApp use remained stable with a slight increase during lockdown. This network maintained the high level of acceptance it already had due to students' perceptions of usefulness, availability of learning support, motivation and possibilities to connect with peers, as was the case in other geographical areas (Mulyono et al., 2021).

However, other sites such as YouTube saw a decline in their use. This could be because it has a less social and more unidirectional dimension (Köseoglu & Köksal, 2018). We should not forget the need for social relations that developed during lockdown. Twitter is a similar case, as it ceased to fulfil its function of disseminating in-person events or initiatives (Köseoğlu & Köksal, 2018). In the same vein, other papers such as that by Ourique et al. (2021) revealed that the aspiring teachers in their study did not find a favourable response to their expectations of synchronous encounters on YouTube, which is blamed on a lack of experience.

The open question about the use of GSNSs enabled us to perform a qualitative analysis shown in Fig. 1, which showed co-occurrences in the use of GSNSs in educational settings. Accordingly, we analysed the 12 categories displayed.

The results we obtained are in line with previous papers as they reflect intensive use of GSNSs by pre-service teachers, something that has been apparent in the entire sample (Keles, 2018). As noted above, one of the main ways that pre-service teachers use GSNSs for educational purposes is to search for teaching resources, without contributing their own.

The students in the sample also identified group work and the creation of environments as important aspects. Establishing how social networking sites are used by the main figures in the teaching–learning process and what benefits they derive from this use has been a constant in academic literature (Santoveña-Casal, 2012). Authors like Serrano Puche (2013) have focused on the idea that these virtual interactions and relationships are as real as the ones established in the physical world. Accordingly, it is logical to think that classrooms cannot be conceived without establishing these work networks (Callaghan & Bower, 2012). Pre-service teachers, as this study shows, carry out their learning process on the basis of different experiences, thus improving their capabilities. This is in line with the research carried out by Kearney et al. (2020).

As for the issue of following news relating to the authorities and staying informed, this is one of the reported uses, although not one of the fundamental uses. This is in line with the findings of Wickramanayake and Jika (2018). Indeed, Blumenreich and Jaffe-Walter (2015) noted the usefulness of social media for working on educational policies.

Finally, our analysis of the responses revealed numerous references relating to the motivational dimension of digital competence. This motivation is very important for understanding what leads students to include GSNSs in their learning processes and in their future work as teachers. This coincides with the papers discussed in the theoretical framework (Minor et al., 2019).

## Findings, Limitations, and Implications

In conclusion, and as has been noted above, this research shows that teachers tend to use WhatsApp, YouTube, and Instagram during their initial training. There are clear differences in their use of such, depending on whether it is for social purposes or as a repository of educational resources. In any case, it reveals a need to make better use of social capital, establish more intense contact networks, greater critical literacy and increase competences so that students can take full advantage of the benefits offered by social networks to develop their own materials and creations and to enter the labour market or academic fields of their interest.

The sample analysed showed that undergraduate students used GSNSs more intensively than postgraduates. This is because of the need to establish educational communities that are stable over time. With regard to the master’s students, as their studies last only one academic year and are of a strictly professional nature, the students' use was less intense and more pragmatic, immediate and occasional, such that students lost interest in establishing communities and their relationships on the networks were less smooth.

Regarding types of university, the students in fully online education displayed a greater need to meet their affective and relational needs through GSNSs. Their educational use is similar to that made at an in-person university, but with a more emotional dimension. In this regard, the sense of “belonging” that can be developed on social networks was revealed to be of high importance and in these universities it was shown to be a factor of satisfaction and, thus, indicating a high predictability of continued use.

In terms of the effects of the situation caused by COVID-19, the target population of this study is not among the groups most affected by the digital divide that was greatly revealed in other areas, however it was possible to identify changes in relationships that were initially temporary, resulting from precariousness and uncertainty. Networks were subsequently established enabling the amplification of the social dimension, to the detriment of networks, with the purpose of disseminating events or simply searching for resources. Not enough time has passed to fully see what uses and effects will be the product of the times when online activity was imposed in education, or what changes have been so profound that they have remained. Nevertheless, it can be affirmed that there have been clear transformations in the use of GSNSs.

In any case, we found that pre-service teachers consume more resources than what they produce, thus missing out on professional opportunities and social capital. However, one of the strong points of the networks analysed was motivation. There is no doubt that times of change are approaching since we live in an ever-changing society and GSNSs reflect this change. Accordingly, pre-service teachers must learn to make the most of all of the advantages and, at the same time, prevent all of the potential problems stemming from social networking sites. That is the only way they will be able to help their future students avoid these problems and reap all the benefits.

The limitations of this study stem from the sample collection strategy and protocol. Potential respondents were invited to take part through online media or through researchers' own professional networks and peer networks. This may result in a respondent bias, as students who are more likely to use social networks may have been attracted to complete the survey. They may even be individuals with a more critical self-perception of their social network activities. In addition, the question of how the use of GSNSs affects academic performance is also a limitation as well as a future research opportunity.

The implications of these findings are related to those previously revealed by a great deal of the scientific literature: there is a need for further research on specific aspects such as the mistrust that the networks produce in their academic use among peers and in the relationships between teachers and students, given that in the latter case they are limited to quite specific experiences. There is also a need to address issues such as how to unite formal, non-formal and informal contexts through GSNSs without causing social overload, overuse, excessive interference in private life or inappropriate attitudes. The research also reveals that institutions need to take part in this change by making communication channels and communities generated through social networks more flexible and promoting them. While it is true that it is precisely the more independent nature and freedom from the constraints of some academic or institution-specific and private networks that arouses interest among students, it would be useful to analyse the way in which resources, personal promotion and assessment systems are conveyed through the networks. Ultimately, the empirical findings of this study support the need to guide education policies and decision-making towards planning, assessing and implementing the use of social networks, either by integrating them into platforms or including them in teaching–learning processes and good assessment practices, especially with regard to teachers in training.

The future research opportunities to be implemented regarding teachers, students and institutions include promoting without exercising excessive control, teaching digital literacy and critical competence, shaping real educational communities, and developing quality educational experiences and products. This study also proposes future lines of research that refer to students’ acceptance of the implementation of digital technology, and of social networks in particular, after COVID-19. The changes described highlight a trend that with the return to normality may show itself to be the new reality of education. In this reality, real-time or non-real time interactions between students will take centre stage. In this way, learning communities may be created and managed on social networks in which teachers may or may not be present. In this sense, studying emotional impact, as well as academic performance, appears to be crucial.

## Data Availability

The datasets generated and/or analysed during the current study are available in the first author’s repository, https://cutt.ly/3WvsdB3.

## References

[CR1] Abreu JL (2020). Tiempos de Coronavirus: La Educación en Línea como Respuesta a la Crisis. Daena: International Journal of Good Conscience.

[CR3] Al-Maatouk Q, Othman MS, Aldraiweesh A, Alturki U, Al-Rahmi WM, Aljeraiwi AA (2020). Task-technology fit and technology acceptance model application to structure and evaluate the adoption of social media in academia. IEEE Access.

[CR4] Al-Rahmi AM, Al-Rahmi WM, Alturki U, Aldraiweesh A, Almutairy S, Al-Adwan AS (2021). Exploring the factors affecting mobile learning for sustainability in higher education. Sustainability.

[CR5] Althunibat A (2015). Determining the factors influencing students’ intention to use m-learning in Jordan higher education. Computers in Human Behavior.

[CR6] Amer B, Amer TS (2018). Use of Pinterest to promote teacher–student relationships in a higher education computer information systems course. Journal of the Academy of Business Education.

[CR7] Annamalai N (2018). How do we know what is happening in WhatsApp: A case study investigating pre-service teachers’ online activity. Malaysian Journal of Learning and Instruction.

[CR9] Barak M (2017). Science teacher education in the twenty-first century: A pedagogical framework for technology-integrated social constructivism. Research in Science Education.

[CR10] Barrón. (2020). *La educación en línea. Transiciones y disrupciones en Educación y pandemia, una visión académica*. Instituto de Investigaciones sobre la Universidad y la Educación de la Universidad Nacional Autónoma de México.

[CR11] Barry DS, Marzouk F, Chulak-Oglu K, Bennett D, Tierney P, O'Keeffe GW (2016). Anatomy education for the YouTube generation. Anatomical Sciences Education.

[CR12] Becerra MT, Martín F (2015). Visión de las plataformas virtuales de enseñanza y las Redes Sociales por los usuarios estudiantes universitarios. Un estudio descriptive. Píxel-Bit. Revista de Medios y Educación.

[CR13] Blumenreich M, Jaffe-Walter R (2015). Social media illuminates: Some truths about school reform. Phi Delta Kappan.

[CR14] Brouwer J, Downey C, Bokhove C (2020). The development of communication networks of pre-service teachers on a school-led and university-led programme of initial teacher education in England. International Journal of Educational Research.

[CR15] Callaghan N, Bower M (2012). Learning through social networking sites. The critical role of the teacher. Educational Media International.

[CR16] Camilleri MA (2021). Evaluating service quality and performance of higher education institutions: A systematic review and a post-COVID-19 outlook. International Journal of Quality and Service Sciences.

[CR18] Camilleri MA, Camilleri AC (2021). The acceptance of learning management systems and video conferencing technologies: Lessons learned from COVID-19. Technology, Knowledge and Learning.

[CR17] Camilleri, M. A., & Camilleri, A. C. (2022). Remote learning via video conferencing technologies: Implications for research and practice. *Technology in Society, 68*. https://www.sciencedirect.com/science/article/pii/S0160791X22000227 10.1016/j.techsoc.2022.101881 PMC8743284 35034998

[CR19] Canaleta X, Vernet D, Vicent L, Montero JA (2014). Master in teacher training: A real implementation of active learning. Computers in Human Behavior.

[CR22] Carpenter J, Krutka D (2015). Engagement through microblogging: Educator professional development via Twitter. Professional Development in Education.

[CR135] Carpenter, J. P., Tur, G., & Marín, V. I. (2016). What do US and Spanish pre-service teachers think about educational and professional use of Twitter?. A comparative study. *Teaching and Teacher Education*, *60*, 131–143. 10.1016/j.tate.2016.08.011

[CR21] Carpenter JP, Harvey S (2019). “There's no referee on social media”: Challenges in educator professional social media use. Teaching and Teacher Education.

[CR20] Carpenter JP, Kimmons R, Short CR, Clements K, Staples ME (2019). Teacher identity and crossing the professional–personal divide on Twitter. Teaching and Teacher Education.

[CR23] Carpenter J, Tani T, Morrison S, Keane J (2020). Exploring the landscape of educator professional activity on Twitter: An analysis of 16 education-related Twitter hashtags. Professional Development in Education.

[CR24] Cavus N, Sani AS, Haruna Y, Lawan AA (2021). Efficacy of social networking sites for sustainable education in the era of COVID-19: A systematic review. Sustainability.

[CR25] Chamarro, A. (2020). Impacto psicosocial del COVID-19: algunas evidencias, muchas dudas por resolver. *Aloma, 38*(1), 9–12. https://www.raco.cat/index.php/Aloma/article/view/371868/465459

[CR26] Chang-Kredl S, Colannino D (2017). Constructing the image of the teacher on Reddit: Best and worst teachers. Teaching and Teacher Education.

[CR27] Chen SY, Kuo HY, Hsieh TC (2019). New literacy practice in a Facebook group: The case of a residential learning community. Computers and Education.

[CR28] Chintalapati N, Daruri VSK (2017). Examining the use of YouTube as a Learning Resource in higher education: Scale development and validation of TAM model. Telematics and Informatics.

[CR29] Colás, P., Conde, J., & Martín, A. (2015). Las Redes Sociales en la enseñanza universitaria: Aprovechamiento didáctico del capital social e intelectual. *Revista Interuniversitaria de Formación del Profesorado, 83*, 105–116. https://www.redalyc.org/pdf/274/27443659008.pdf

[CR31] Davis, F. D. (1986). A technology acceptance model for empirically testing new end-user information systems: Theory and results*.* Unpublished Doctoral Dissertation, Massachusetts Institute of Technology.

[CR32] Davis FD (1989). Perceived usefulness, perceived ease of use, and user acceptance. MIS Quarterly.

[CR33] Dennen VP, Choi H, Word K (2020). Social media, teenagers, and the school context: A scoping review of research in education and related fields. Educational Technology Research and Development.

[CR130] Dermentzi, E., Papagiannidis, S., Toro, C. O., & Yannopoulou, N. (2016). Academic engagement: Differences between intention to adopt social networking sites and other online technologies. *Computers in Human Behavior*, *61*, 321–332. 10.1016/j.chb.2016.03.019

[CR34] Durak HY (2019). Examining the acceptance and use of online social networks by preservice teachers within the context of unified theory of acceptance and use of technology model. Journal of Computing in Higher Education.

[CR35] Ekoç A (2020). No teacher is an island: Technology-assisted personal learning network (PLN) among English language teachers in Turkey. Interactive Learning Environments.

[CR36] Fishbein, M., & Ajzen, I. (1975). *Belief, attitude, intention, and behavior: An introduction to theory and research*. Addison-Wesley.

[CR37] Fondevila JF, Marqués J, Mir P, Polo M (2019). Usos del WhatsApp en el estudiante universitario español. Pros y contras. Revista Latina de Comunicación Social.

[CR38] Fuchs, C. (2019). Critical incidents and cultures-of-use in a Hong Kong–Germany telecollaboration. *Language Learning and Technology, 23*(3), 74–97. http://hdl.handle.net/10125/44697

[CR39] García-Peñalvo FJ, Corell A, Abella-García V, Granded M (2020). La evaluación online en la educación superior en tiempos de la COVID-19. Education in the Knowledge Society.

[CR40] Gil-Hernández R, Calderón-Garrido D (2021). Implicaciones de la Teoría de Usos y Gratificaciones en las prácticas mediadas por redes sociales en el ámbito educativo. Una revisión sistemática de la literature. Aloma. Revista de Psicología, Ciències de l'educació i de l'esport.

[CR41] Gil-Fernández R, León-Gómez A, Calderón-Garrido D (2021). Influence of COVID on the educational use of social media by students of teaching degrees. Education in the Knowledge Society.

[CR42] Goodyear VA, Casey A, Kirk D (2014). Tweet me, message me, like me: Using social media to facilitate pedagogical change within an emerging community of practice. Sport, Education and Society.

[CR43] Goodyear VA, Parker M, Casey A (2019). Social media and teacher professional learning communities. Physical Education and Sport Pedagogy.

[CR44] Greene K (2017). Teacher blogs and education policy in a publicly private world: Filling the gap between policy and practice. Learning, Media and Technology.

[CR45] Greenhalgh SP, Rosenberg JM, Staudt Willet KB, Koehler MJ, Akcaoglu M (2020). Identifying multiple learning spaces within a single teacher-focused Twitter hashtag. Computers and Education.

[CR46] Greenhow, C., & Chapman, A. (2020). Social distancing meet social media: Digital tools for connecting students, teachers, and citizens in an emergency. *Information and Learning Sciences, 121*, 7/8. https://www.emerald.com/insight/2398-5348.htm

[CR47] Hashim AK, Carpenter JP (2019). A conceptual framework of teacher motivation for social media use. Teachers College Record.

[CR48] Hasiloglu MA, Calhan HS, Ustaoglu ME (2020). Determining the views of the secondary school science teachers about the use of social media in education. Journal of Science Education and Technology.

[CR49] Hatzipanagos S, John BA (2017). Do Institutional social networks work? Fostering a sense of community and enhancing learning. Technology, Knowledge and Learning.

[CR50] Hatzipanagos, S., & Warburton, S. (Eds.). (2009). * Social software and developing community ontologies*. IGI Global.

[CR51] Hodges, C., Moore, S., Lockee, B., Trust, T., & Bond, A. (2020). The difference between emergency remote teaching and online learning*. Educause Review*. https://er.educause.edu/articles/2020/3/the-difference-between-emergency-remote-teaching-and-online-learning

[CR52] Imlawi J, Gregg D, Karimi J (2015). Student engagement in course-based social networks: The impact of instructor credibility and use of communication. Computers and Education.

[CR53] Jan SK, Vlachopoulos P (2019). Social network analysis: A framework for identifying communities in higher education online learning. Technology, Knowledge and Learning.

[CR54] Kamalodeen VJ, Jameson-Charles M (2016). A mixed methods research approach to exploring teacher participation in an online social networking website. International Journal of Qualitative Methods.

[CR55] Katz, E., Blumler, J. G., & Gurevitch, M. (1974). *Utilization of mass communication by the individual*. In J. G. Blumler & E. Katz (Eds.), *The use of mass communications: Current perspectives on gratifications research*. University of Chicago Press.

[CR56] Kearney M, Maher D, Pham L (2020). Investigating pre-service teachers' informally-developed online professional learning networks. Australasian Journal of Educational Technology.

[CR57] Keles E (2018). Use of Facebook for the Community Services Practices course: Community of inquiry as a theoretical framework. Computers and Education.

[CR59] Khoza SB (2021). Can teachers’ identities come to the rescue in the fourth industrial revolution?. Technology, Knowledge and Learning..

[CR131] Kim, W., & Malek, K. (2018). Social networking sites versus professional networking sites: Perceptions of hospitality students. *Journal of Human Resources in Hospitality and Tourism*, *17*(2), 200–221. 10.1080/15332845.2017.1340763

[CR132] Dhir, A., Khalil, A., Lonka, K., & Tsai, C. C. (2017). Do educational affordances and gratifications drive intensive Facebook use among adolescents? *Computers in Human Behavior*, *68*, 40–50. 10.1016/j.chb.2016.11.0140

[CR133] Ghareb, M. I., Ahmed, Z. A., & Ameen, A. A. (2018). The role of learning through social network in higher education in KRG. *International Journal of Scientific and Technology Research*, *7*(5), 20–27. https://www.ijstr.org/final-print/may2018/The-Role-Of-Learning-Through-Social-Network-In-Higher-Education-In-Krg.pdf

[CR58] Köseoğlu P, Köksal MS (2018). An analysis of prospective teachers’ perceptions concerning the concept of “Social Media” through metaphors. Eurasia Journal of Mathematics, Science and Technology Education.

[CR60] Krutka DG, Bergman DJ, Flores R, Mason K, Jack AR (2014). Microblogging about teaching: Nurturing participatory cultures through collaborative online reflection with pre-service teachers. Teaching and Teacher Education.

[CR61] Lantz-Andersson A, Lundin M, Selwyn N (2018). Twenty years of online teacher communities: A systematic review of formally-organized and informally-developed professional learning groups. Teaching and Teacher Education.

[CR63] Marchetti E, Valente A (2018). Interactivity and multimodality in language learning: The untapped potential of audiobooks. Universal Access in the Information Society.

[CR64] Marín VI, Carpenter JP, Tur G (2020). Pre-service teachers’ perceptions of social media data privacy policies. British Journal of Educational Technology.

[CR65] Matzat U (2013). Do blended virtual learning communities enhance teachers' professional development more than purely virtual ones? A large scale empirical comparison. Computers and Education.

[CR66] McCarthy J (2017). Enhancing feedback in higher education: Students’ attitudes towards online and in-class formative assessment feedback models. Active Learning in Higher Education.

[CR67] McGarr O, McDonagh A (2020). Exploring the digital competence of pre-service teachers on entry onto an initial teacher education programme in Ireland. Irish Educational Studies.

[CR68] Mingle, J., & Adams, M. (2015). Social media network participation and academic performance in senior high schools in Ghana. *Library Philosophy and Practice,**2015*(1), 1286. http://digitalcommons.unl.edu/libphilprac/1286

[CR69] Minor, E. C., Saw, G. K., Frank, K. A., Schneider, B., & Torphy, K. T. (2019). External contextual factors and teacher turnover: The case of Michigan high schools. *Teachers College Record, 121*(11). https://eric.ed.gov/?id=EJ1261809

[CR70] Mnkandla E, Minnaar A (2017). The use of social media in e-learning: A metasynthesis. The International Review of Research in Open and Distributed Learning.

[CR71] Mulyono H, Suryoputro G, Jamil SR (2021). The application of WhatsApp to support online learning during the COVID-19 pandemic in Indonesia. Heliyon.

[CR73] Nwosu AC, Monnery D, Reid VL, Chapman L (2017). Use of podcast technology to facilitate education, communication and dissemination in palliative care: The development of the AmiPal podcast. BMJ Supportive and Palliative Care.

[CR74] Ourique MLH, Lage LD, Bueno TI (2021). Being the presence in the absence: Teacher's (self) education under the COVID-19 pandemic. Humanidades and Inovacao.

[CR75] Park N, Lee KM, Cheong PH (2007). University instructors’ acceptance of electronic courseware: An application of the technology acceptance model. Journal of Computer-Mediated Communication.

[CR76] Park SY, Nam MW, Cha SB (2012). University students’ behavioral intention to use mobile learning: Evaluating the technology acceptance model. British Journal of Educational Technology.

[CR77] Peña MA, Rueda E, Pegalajar MC (2018). Posibilidades didácticas de las Redes Sociales en el desarrollo de competencias de educación superior: percepciones del alumnado. Pixel-Bit. Revista de Medios y Educación.

[CR78] Pérez-Garcías A, Tur G, Darder-Mesquida A, Marín VI (2020). Reflexive skills in teacher education: A tweet a week. Sustainability.

[CR79] Phua J, Jin SV, Kim JJ (2017). Gratifications of using Facebook, Twitter, Instagram, or Snapchat to follow brands: The moderating effect of social comparison, trust, tie strength, and network homophily on brand identification, brand engagement, brand commitment, and membership intention. Telematics and Informatics.

[CR80] Prestridge S, Tondeur J, Ottenbreit-Leftwich AT (2019). Insights from ICT-expert teachers about the design of educational practice: The learning opportunities of social media. Technology, Pedagogy and Education.

[CR81] Rathnayake C, Winter JS (2018). Carrying forward the uses and Grats 2.0 agenda: An affordance-driven measure of social media uses and gratifications. Journal of Broadcasting and Electronic Media.

[CR134] Raza, S. A., Qazi, W., Umer, B., & Khan, K. A. (2020). Influence of social networking sites on life satisfaction among university students: A mediating role of social benefit and social overload. *Health Education*, *120*(2), 141–164. 10.1108/HE-07-2019-003

[CR82] Rensfeldt AB, Hillman T, Selwyn N (2018). Teachers “liking” their work? Exploring the realities of teacher Facebook groups. British Educational Research Journal.

[CR83] Rogers, H., et al. (2020). *The COVID-19 Pandemic: Shocks to education and policy responses.* The World Bank. http://documents.worldbank.org/curated/en/804001590734163932/pdf/The-COVID-19-Pandemic-Shocks-to-Education-and-Policy-Responses.pdf

[CR86] Saini Ch, Abraham J (2019). Modeling educational usage of social media in pre-service teacher education. Journal of Computing in Higher Education.

[CR87] Sánchez JA, Arrazola J, Calderón D (2017). The DIYLab project (Do It Yourself in Education: Expanding Digital Competence to Foster Student Agency and Collaborative Learning). Cultura y Educación.

[CR89] Santisteban A, Díez-Bedmar MC, Castellví J (2020). Critical digital literacy of future teachers in the Twitter Age. Culture and Education.

[CR90] Santoveña-Casal S (2012). Calidad de la metodología didáctica por medio de entornos virtuales de aprendizaje en la formación de agentes educativos. Contextos Educativos: Revista de Educación.

[CR91] Sawyer A, Dick L, Shapiro E, Wismer T (2019). The top 500 mathematics pins: An analysis of elementary mathematics activities on Pinterest. Journal of Technology and Teacher Education.

[CR92] Schroeder S, Curcio R, Lundgren L (2019). Expanding the learning network: How teachers use Pinterest. Journal of Research on Technology in Education.

[CR93] Serrano Puche J (2013). Vidas conectadas: Tecnología digital, interacción social e identidad. Historia y Comunicación Social.

[CR94] Simpson A (2016). Designing pedagogic strategies for dialogic learning in higher education. Technology, Pedagogy and Education.

[CR95] Sinnema C, Daly AJ, Liou YH, Rodway J (2020). Exploring the communities of learning policy in New Zealand using social network analysis: A case study of leadership, expertise, and networks. International Journal of Educational Research.

[CR96] Somerville, J. A. (2008). *Effective use of the Delphi process in research: Its characteristics, strengths and limitations*. Corvallis. http://jasomerville.com/wp-content/uploads/2011/08/DelphiProcess080617b.pdf

[CR97] Staudt Willet KB (2019). Revisiting how and why educators use Twitter: Tweet types and purposes in #Edchat. Journal of Research on Technology in Education.

[CR98] Suárez, B. (2018). Whatsapp: su uso educativo, ventajas y desventajas. *Revista de Investigación en Educación, 16*(2), 121–135. http://reined.webs.uvigo.es/index.php/reined/article/view/342

[CR99] Szeto E, Cheng AYN, Hong JC (2016). Learning with social media: How do preservice teachers integrate YouTube and social media in teaching?. The Asia-Pacific Education Researcher.

[CR100] Thompson J, Hagenah S, Lohwasser K, Laxton K (2015). Problems without ceilings: How mentors and novices frame and work on problems-of-practice. Journal of Teacher Education.

[CR101] Torphy, K. T., Brandon, D. L., Daly, A. J., Frank, K. A., Greenhow, C., Hu, S., & Rehm, M. (2020a). Social media, education, and digital democratization*. Teachers College Record, 122*(6). https://academicworks.cuny.edu/cgi/viewcontent.cgi?article=1109&context=cc_etds_theses

[CR103] Torphy K, Hu S, Liu Y, Chen Z (2020). Teachers turning to teachers: Teacherpreneurial behaviors in social media. American Journal of Education.

[CR102] Torphy K, Liu Y, Hu S, Chen Z (2020). Sources of professional support: Patterns of teachers’ curation of instructional resources in social media. American Journal of Education.

[CR104] Trust T (2017). Motivation, empowerment, and innovation: Teachers' beliefs about how participating in the Edmodo math subject community shapes teaching and learning. Journal of Research on Technology in Education.

[CR105] Tur G, Marín V, Carpenter J (2017). Using Twitter in higher education in Spain and the USA. Comunicar Media Education Research Journal.

[CR106] Tyrer C (2019). Beyond social chit chat? Analysing the social practice of a mobile messaging service on a higher education teacher development course. International Journal of Educational Technology in Higher Education.

[CR107] Valencia-Ortiz R, Cabero-Almenara J, Garay-Ruiz U (2020). Influencia del género en el uso de redes sociales por el alumnado y profesorado. Campus Virtuales.

[CR108] Venkatesh V, Thong JY, Xu X (2012). Consumer acceptance and use of information technology: Extending the unified theory of acceptance and use of technology. MIS Quarterly.

[CR109] Wickramanayake L, Jika SM (2018). Social media use by undergraduate students of education in Nigeria: A survey. The Electronic Library.

[CR111] Yakin I, Tinmaz H (2015). Theoretical guidelines for the utilization of instructional social networking websites. Turkish Online Journal of Distance Education.

